# Dispatches from the neighborhood watch: Using citizen science and field survey data to document color morph frequency in space and time

**DOI:** 10.1002/ece3.6006

**Published:** 2020-01-16

**Authors:** Richard M. Lehtinen, Brian M. Carlson, Alyssa R. Hamm, Alexis G. Riley, Maria M. Mullin, Weston J. Gray

**Affiliations:** ^1^ The College of Wooster Department of Biology Wooster OH USA

**Keywords:** citizen science, color polymorphism, genetic drift, *Sciurus*, selection, spatial patterns

## Abstract

Heritable color polymorphisms have a long history of study in evolutionary biology, though they are less frequently examined today than in the past. These systems, where multiple discrete, visually identifiable color phenotypes co‐occur in the same population, are valuable for tracking evolutionary change and ascertaining the relative importance of different evolutionary mechanisms. Here, we use a combination of citizen science data and field surveys in the Great Lakes region of North America to identify patterns of color morph frequencies in the eastern gray squirrel (*Sciurus carolinensis*). Using over 68,000 individual squirrel records from both large and small spatial scales, we identify the following patterns: (a) the melanistic (black) phenotype is often localized but nonetheless widespread throughout the Great Lakes region, occurring in all states and provinces sampled. (b) In Ohio, where intensive surveys were performed, there is a weak but significantly positive association between color morph frequency and geographic proximity of populations. Nonetheless, even nearby populations often had radically different frequencies of the melanistic morph, which ranged from 0% to 96%. These patterns were mosaic rather than clinal. (c) In the Wooster, Ohio population, which had over eight years of continuous data on color morph frequency representing nearly 40,000 records, we found that the frequency of the melanistic morph increased gradually over time on some survey routes but decreased or did not change over time on others. These differences were statistically significant and occurred at very small spatial scales (on the order of hundreds of meters). Together, these patterns are suggestive of genetic drift as an important mechanism of evolutionary change in this system. We argue that studies of color polymorphism are still quite valuable in advancing our understanding of fundamental evolutionary processes, especially when coupled with the growing availability of data from citizen science efforts.

## Graphical abstract

This paper describes the background, research questions and design of the Research Training Group “ConFoBi” (Conservation of Forest Biodiversity in Multiple‐use Landscapes of Central Europe). ConFoBi focusses on the effectiveness of retention forestry, that is, retaining structural elements of old forests within a production forest matrix, and combines ecological studies on forest biodiversity with social and economic studies of biodiversity conservation across multiple spatial scales. The aim of ConFoBi is to assess whether and how structural retention measures are appropriate for the conservation of forest biodiversity in multiple‐use landscapes of temperate Europe.

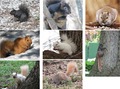

## 1 INTRODUCTION

Heritable genetic polymorphisms are an important source of the variation that is the raw material of evolutionary change. The empirical patterns exhibited by genetic polymorphisms and the processes influencing their change in frequency over space and time have long been of fundamental interest in evolutionary biology (Crow & Kimura, 1970; Majerus, 1998). In particular, heritable color polymorphisms have been an important tool to address fundamental evolutionary questions because of their discrete and visually conspicuous nature (Mayr & Provine, 1998). Because of this advantage, heritable color polymorphisms have been used to study a wide variety of evolutionary phenomena including sexual selection (Svensson, Abbott, Gosden, & Coreau, 2009), the genetic and regulatory basis of phenotypes (Andrade et al., 2019), frequency dependence (Svensson, Abbott, & Härdling, 2005), gene flow (Bittner & King, 2003), and speciation (Corl, Davis, Kuchta, & Sinervo, 2010; McLean & Stuart‐Fox, 2014).

Perhaps, the most important use of color polymorphisms, however, has been to illustrate the power of natural selection. For example, Hoekstra, Drumm, and Nachman (2004) demonstrated the selective advantage that uniformly dark (melanistic) pocket mice have on dark substrates compared with wild type individuals typically found on lighter substrates. Other examples include light‐colored beach mice (Hoekstra, Hirschmann, Bundey, Insel, & Crossland, 2006), melanistic ladybird beetles (Creed, 1975) and color polymorphic land snails (Clarke & Murray, 1962; Jones, Leith, & Rawlings, 1977). These and other studies have often found evidence of clines in color morph frequency over space which have been interpreted as a signature of spatially variable selection. This is especially evident in the most well‐studied example of the action of natural selection using a color polymorphic species, the peppered moth (*Biston betularia*). This example is especially illuminating because of its large spatial (hundreds of localities on two continents) and temporal (decades long) replication (Clarke, Grant, Clarke, & Asami, 1994; Kettlewell, 1955, 1958; Majerus, 2009). The strength of the peppered moth work is a limitation of many other field studies, which are often poorly replicated over time and space (Svensson, 2017).

Since there are a number of compelling examples of the action of selection on the frequency of color morphs, it is often assumed that geographic variation in such traits has a selective origin (McLean & Stuart‐Fox, 2014). However, there are other possible explanations, chief among them is genetic drift. Genetic drift has long been looked down upon as a relatively unimportant mechanism of evolutionary change, except under a narrow range of circumstances (small population sizes, neutral substitutions; Gould, 2002; Lenormand, Roze, & Rousset, 2009). And yet, a number of empirical studies of color polymorphic species have identified drift as an important mechanism of change, producing mosaic rather than clinal color morph frequency distributions in space (e.g., McLean & Stuart‐Fox, 2014; Reillo & Wise, 1988; Runemark, Hansson, Pafilis, Valakos, & Svensson, 2010; Van Gossum, Beirinckx, Forbes, & Sherratt, 2007). Assessing the relative importance of selection, gene flow and drift in natural systems are of fundamental importance to fully understand the evolutionary process (Nachman, Hoekstra, & D'Agostino, 2003; Raymond, Chevillon, Guillemaud, Lenormand, & Pasteur, 1998; Reznick, Shaw, Rodd, & Shaw, 1997). Nonetheless, these phenomena are challenging to track in real time. Herein, lies the advantage of color polymorphisms: They are easily assessed visually and, for common species, large sample sizes allow frequency estimates to be made with relatively high precision (Svensson, 2017). Thus, geographically variable color polymorphic species are good models for studying the interaction between drift, selection, and gene flow (McLean & Stuart‐Fox, 2014).

Technological advances and the rise of citizen science efforts (Chandler et al., 2017) have made it possible to assess the spatial and temporal patterns of color morph frequencies in unprecedented detail. Common and widespread species such as the eastern gray squirrel (*Sciurus carolinensis*) are particularly well suited to such studies. This species is conspicuousness, easy to identify and often inhabits environments that are frequented by humans. While there is individual, seasonal and geographic variation, the typical eastern gray squirrel body coloration is gray with a white belly (Whitaker & Hamilton, 1998). However, all black (melanistic) individuals are also present in some populations and they can be quite common (Brayton, 1882; Burt, 1957). White morph individuals are also known in some areas (leucistic and albino; Barkalow & Shorten, 1973). While some work has assessed physiological (Ducharme, Larochelle, & Richard, 1989) and genetic differences (McRobie et al., 2014; McRobie, Thomas, & Kelly, 2009; Shorten, 1945) among gray squirrel color morphs, only one study has quantitatively assessed the spatial distribution of the color morphs. Using citizen science data, Gibbs, Buff, and Cosentino (2019) found a positive relationship between urban land use and the occurrence of the black color morph and a negative relationship between black color morph occurrence and increasing temperature. This latter result supports the anecdotal observations of earlier authors who noted that the black morph is especially common in the northern portion of their range (Banfield, 1974; Gustafson & VanDruff, 1990). This could point to a selective advantage of the black morph in cold climates due to greater heat absorption or heat generating capacity (Ducharme et al., 1989). Gibbs et al. (2019) also suggested that spatially variable selection (selection against the black morph in nonurban areas due to human hunting and selection against the gray morph in urban areas due to road mortality) explains their observed geographic variation in color morph frequencies.

To test these ideas, we used citizen science data from the Great Lakes region of the United States and Canada and our own field surveys at three spatial scales to expand the spatial and temporal scope of studies in this system. Specifically, we test the following hypotheses. (a) We test the reliability of citizen science data by statistically comparing citizen science estimates of color morph frequency and distribution to our own. We predict no difference between datasets. (b) We examine the frequency of the black morph on a large spatial scale to test the hypothesis of temperature as a potential selective agent favoring the black morph in colder regions. We predict a positive linear relationship between black morph frequency and increasing latitude and no relationship to longitude. (c) We examine spatial occurrence and frequency patterns of the color morphs in a smaller region to identify clinal or mosaic geographic patterns that would be suggestive of the action of selection or drift. We predict a clinal spatial pattern indicating the existence of spatially variable selection. (d) We use a long time series of observations at one locality to provide the first data on morph frequency stability or change over time in this system. We predict stability in color morph frequencies over time.

## 2 MATERIALS AND METHODS

To assess the spatial distribution of the melanistic phenotype in the Great Lakes region, we used both data from our own field surveys as well as citizen science data. The citizen science data came from two sources: The iNaturalist online database and hunter records compiled by the Ohio Natural History Database.

### 2.1 iNaturalist records

We downloaded gray squirrel (*S. carolinensis*) records from the http://iNaturalist.org citizen science database including all records up through June 2018 from the following U.S. states and Canadian provinces in the Great Lakes watershed: Illinois, Indiana, Michigan, Minnesota, Ohio, Pennsylvania, New York, Wisconsin, Ontario, and Quebec. Each record includes a digital photograph and the time, date and locality information submitted by a user via smartphone. The oldest record was from 2005 but the large majority were from 2015 onwards. We culled duplicate records, those with misidentified animals and those with other obvious errors. The remaining records were manually checked for the color morph of the squirrel in the photograph. These were recorded as gray, melanistic, or white based on pelage characteristics. Photographs in which pelage color could not be confidently determined (e.g., in heavy shade) were discarded. If there was more than one individual in a photograph, each individual was counted separately. Estimates of color morph frequency should be interpreted cautiously from these data as citizen scientists may be more likely to submit observations perceived as novel (black or white squirrels) than the typical coloration pattern. We organized these data by county (for the US records) and by census divisions (for Canadian provinces) to create a map of occurrence of the melanistic morph using ArcGIS.

### 2.2 Ohio natural history database records

We also acquired records from the Ohio Natural History Database provided by squirrel hunters from the 2012, 2013, and 2014 hunting seasons (September through January) in Ohio. In these voluntary reports, hunters self‐reported the number of gray and black morph gray squirrels seen while hunting as well as the date and location of the observations. Unlike the iNaturalist records, no photographs were available to confirm observations. As above, we organized these records by county to create maps of occurrence of the melanistic morph.

### 2.3 Regional squirrel surveys

To provide information on not just the occurrence but also an initial estimate of the relative frequency of the color morphs, we conducted squirrel surveys in 59 localities in twenty‐two counties in Ohio (USA) from June 2018 through July 2019 during daylight hours. These sites were mostly focused in north‐central, central, and northeastern Ohio (~18,000 km^2^, with a few outside these areas). Sampling often consisted of a single site visit. Of these 59 localities, 55 were urban and four were nonurban forests. In urban localities, we used a combination of car‐based and walking surveys in public parks, residential areas, cemeteries, and college campuses to visually observe free‐living squirrels and assess their pelage color. One to four observers were used on each survey and care was taken to avoid double‐counting. Walking surveyors used a normal walking pace (~1.5 m/s), surveyors from a vehicle drove at approximately 20 km/h. We attempted to detect a minimum of 25 eastern gray squirrels at each locality to provide an initial estimate of color morph frequency. At three localities (Fern Valley Field Station in Holmes County, The Wilderness Center in Stark County and Wooster Memorial Park in Wayne County) that were nonurban forests, we used a combination of walking surveys as described above and motion‐activated wildlife cameras (StealthCam LLC, model STC‐G42NG) to detect squirrels. Three wildlife cameras were deployed at each site between May and November, positioned at least 200 m apart at approximately 0.7 m above ground level on trees. Cameras were deployed for an average of 208 camera‐days at each site (range: 128–255) and were moved at least once during the study period to more fully sample each squirrel population. Photographs were downloaded and the proportion of images of black versus gray morph squirrels along with the observations from walking surveys was used as an estimate of color morph frequency in these populations.

In 2019, we also more intensively surveyed 16 localities (12 urban, four forest) in the greater Wooster area (~2,700 km^2^) to obtain more precise estimates of the frequency of melanism in these populations. These 16 sites were visited on two to twenty‐two occasions (median: 4) between May and December until a minimum of 100 squirrel color morph observations were acquired in each locality. As above, for urban sites a combination of walking and car‐based surveys were used; for forested sites a combination of walking surveys and wildlife camera deployments were used.

### 2.4 Wooster squirrel surveys

To assess color morph frequency at an even smaller spatial scale and to assess potential changes in color morph frequency over time, we performed squirrel surveys at a single locality over an extended period. One observer (RML), conducted walking surveys in the city of Wooster (Wayne Co., 40.81°N, 81.94°W) along seven predetermined routes for approximately eight and a half years. Each of these routes followed streets in residential neighborhoods adjacent to the College of Wooster campus and Cornerstone Elementary school and are situated along parallel or perpendicular streets (study area: ~0.33 km^2^). Each route was walked at a normal walking pace (~1.5 m/s). During each survey, the number and color morph of gray squirrels observed were tallied. Surveys began with route 1 on July 22, 2010 with routes 2 through 7 being added on April 1, 2013. During this period, we performed 5,166 squirrel surveys, an average of approximately 1.7 surveys per day (median 2, range 0–6). Squirrel surveys were carried out during daylight hours in all weather until 31 December 2018. Special effort was made to make sure that all routes were sampled with similar frequency among seasons and times of day to guard against potential bias. As these observations include counting many of the same individual squirrels repeatedly at different times, these observations are not independent of one another. To address concerns over nonindependence, we only used the aggregated annual totals for each route in analyses (see below).

### 2.5 Statistical analyses

To explore relationships between latitude, longitude, and color morph frequency from the citizen science data (using only localities with *n* > 50), we assessed linear, logarithmic, inverse, quadratic, and cubic relationships between these variables, using the *R* ^2^ value as a measure of fit. To assess whether citizen science data and the field surveys we conducted resulted in different frequency estimates of squirrel melanism in Ohio, we used a generalized linear mixed model (GLMM) with binomial error structure and a logit link function with survey method as a fixed effect and county as a random effect. Unfortunately, the iNaturalist data only had three counties with *n* > 20 in Ohio; therefore, in this analysis we compared only the Ohio Natural Heritage database data and our field survey data (12 counties had *n* > 20 for both methods). To examine potential population‐level differences in the frequency of melanism, we used a similar GLMM with population (specific locality) as a fixed effect using the 2018 survey data from 59 populations. We used another GLMM with the 2019 survey data from the 16 more intensively surveyed populations with population (specific locality) and habitat type (urban or forest) as fixed effects and survey date as a random effect to address nonindependence concerns. To assess whether morph frequencies have changed over time and over very small spatial scales, we used the 8.5 year dataset from Wooster in another similar GLMM. In this analysis, we designated survey route and survey year as fixed effects, and we also examined a route by year interaction term. Before analysis, data were aggregated into annual summary measures for each year‐route combination to address nonindependence concerns. Overdispersion in these analyses was assessed using the ratio of the sum of squared Pearson residuals to the residual degrees of freedom using the *X* ^2^ distribution (Agresti, 2002).

To assess the relationship between geographic distance and similarity of color morph frequency (i.e., spatial autocorrelation) in Ohio, we assembled matrices of all pairwise geographic distances among sites and all Euclidian similarities of color morph frequencies and used a Mantel test to assess the null hypothesis of no relationship (Mantel, 1967) in PC‐ORD version 4.0. Euclidean similarities of color morph frequencies among populations were calculated as the absolute value of the difference in frequency for each pair of populations. For all frequency data presented, we give the 95% confidence interval of the proportion using the Wilson score interval method (following Brown, Cat, and DasGupta (2001)) using scripts at http://epitools.ausvet.com.au/content.php?page=CIProportion). All statistical analyses were conducted with SPSS version 26.0 unless otherwise noted.

## 3 RESULTS

### 3.1 Citizen science data

**Figure 1:**
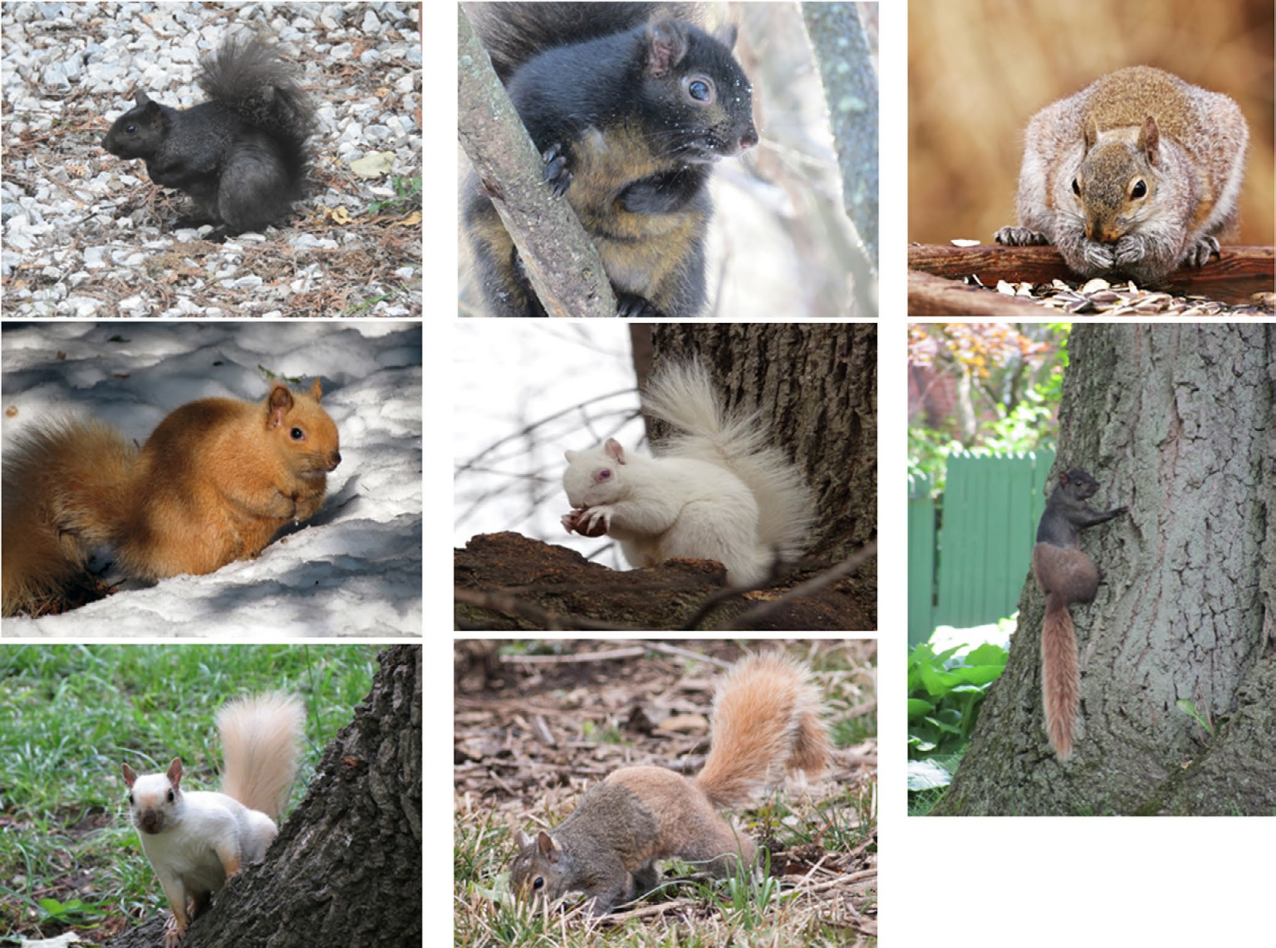
Color variation in eastern gray squirrels (*Sciurus carolinensis*). All photographs were submissions to the http://iNaturalist.org website except that in the lower right (by J. Furey). The top left, top center and bottom right individuals were classified as melanistic. The top right and bottom center individuals were classified as gray. The center and lower left individuals were classified as albino and leucistic, respectively. The middle left image was left unclassified

**Figure 2:**
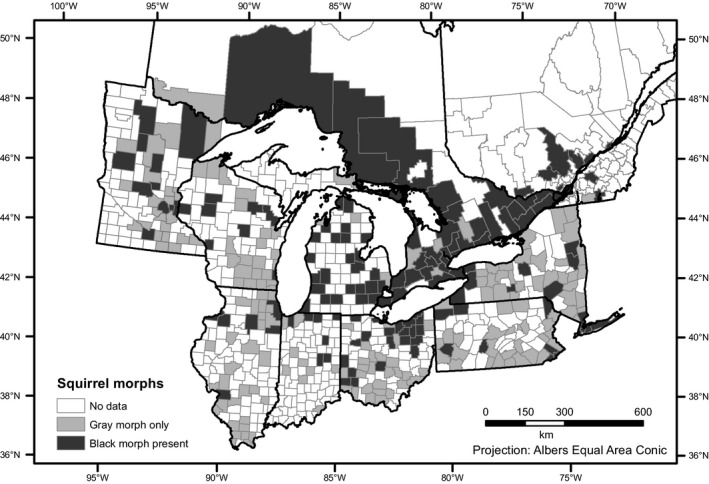
Geographic distribution of the gray and black color morph of the eastern gray squirrel (*Sciurus carolinensis*) in the Great Lakes region of the United States and Canada based on 4,678 citizen science records from http://iNaturalist.org

We used 4,678 records from the iNaturalist database to assess the distribution of gray squirrel melanism in the Great Lakes region. While the sample size varied greatly among states and provinces, all had evidence of the presence of melanistic squirrels (Figure 1). Melanistic squirrels were particularly common in Michigan and Ontario, comprising 89 out of 160 records (56%) and 983 out of 1,461 records (66%), respectively. Melanistic squirrels were present but reported much less frequently in all the remaining states and provinces, representing an average of 15% of all records (range: 5%–28%, Figure 2; Tables [Link], [Link], [Link], [Link], [Link], [Link], [Link], [Link], [Link], [Link]). Interestingly, white squirrels were also documented in every state and province except for Indiana, which also had the lowest sample size. These squirrels with white pelage included both albinos (with pink eyes) as well as leucistic phenotypes (with normal black eyes).

**Figure 3:**
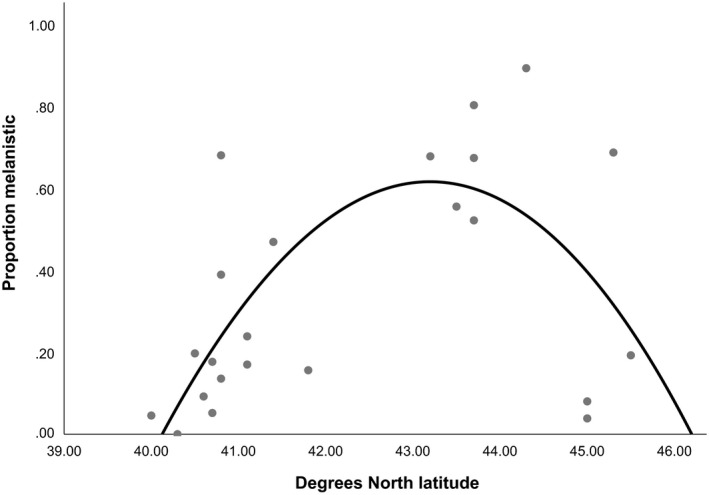
Relationship between the proportion of sampled squirrels that were melanistic (minimum sample size *n* = 50) from 23 localities in the http://iNaturalist.org database and degrees north latitude. The curve indicates a cubic function (*F* = 8.03, *df* = 22, *p* = .03, *R* ^2^ = .445; *y* = −0.001*x* ^3^ + 813.8*x* ^2^ + 2.885*x* − 82.478)

**Figure 4:**
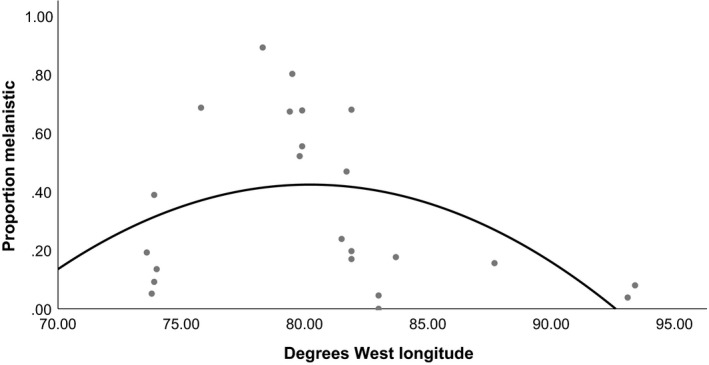
Relationship between the proportion of sampled squirrels that were melanistic (minimum sample size *n* = 50) from 23 localities in the http://iNaturalist.org database and degrees west longitude. The curve indicates a quadratic function (*F* = 2.80, *df* = 22, *p* = .08, *R* ^2^ = .219; *y* = −0.003*x* ^2^ + 0.443*x* − 17.351)

Examination of the relationship between the estimated proportion of melanistic individuals versus latitude showed the best fit to a cubic function (*F* = 8.03, *df* = 22, *p* = .03, *R* ^2^ = .445, Figure 3). Relatively low latitude and relatively high‐latitude localities tended to have a lower proportion of melanistic squirrels while mid‐latitude localities tended to have a higher proportion of melanistic squirrels. The relationship between the estimated proportion of melanistic individuals versus longitude was nonsignificant but showed the best fit to a quadratic function (*F* = 2.80, *df* = 22, *p* = .08, *R* ^2^ = .219, Figure 4) and indicated a peak in melanistic squirrel frequency at mid longitudes and declining at relatively low and high values.

**Figure 5:**
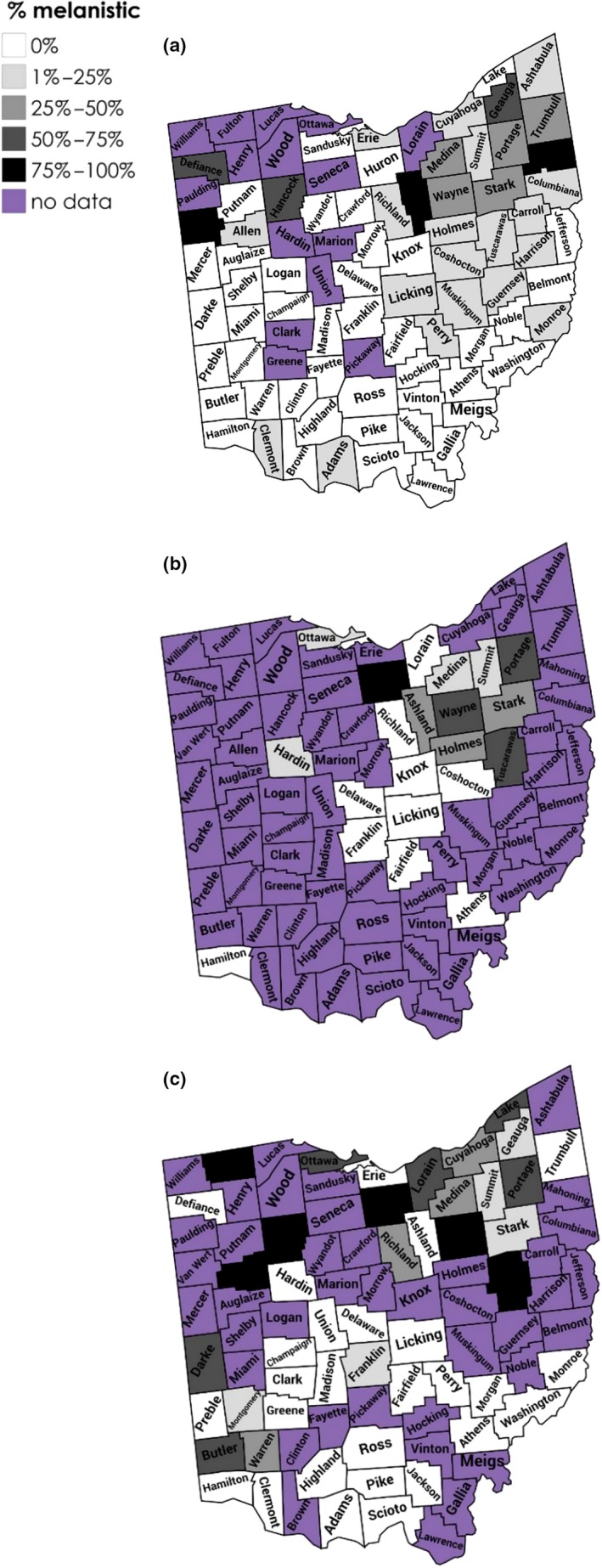
Geographic distribution of the gray and black color morph of the eastern gray squirrel (*Sciurus carolinensis*) in Ohio (USA) based on (a) 7,302 citizen science records in 73 counties from the Ohio Natural History Database, (b) 22,553 observations from our field surveys in 22 counties in 2018 and 2019 and (c) 433 citizen science records in 48 counties from http://iNaturalist.org

The Ohio Natural History Database squirrel hunter records resulted in 7,302 squirrel records from 73 of Ohio's 88 counties with an average sample size per county of 91 (range 1–830). By comparison, the iNaturalist database had records from only 48 of Ohio's 88 counties and an average sample size of nine per county (range 1–110). The squirrel hunter data indicated that melanistic gray squirrels were present in 30 of 73 counties (41%) but were generally rare (<5% of observations) everywhere except for the counties of northeastern Ohio and in Van Wert county in northwestern Ohio (Figure 5, Table S11). No white phenotypes were reported from the squirrel hunter records, despite a much larger sample.

For the twelve counties with *n* > 20 for both the hunter records and our field surveys, we found a highly significant effect of survey method (*F* = 86.9, *df* = 1, *p* < .001) with hunter records reporting lower proportions of melanism, on average, compared to our field surveys (see below). Both similarities and differences are apparent in comparing the spatial distribution of the melanistic phenotype based on the three different sources of data (Figure 5). For example, all data sources identified northeastern Ohio as an area where melanism is common. However, data sources sometimes showed inconsistent occurrence or frequency patterns in some areas (e.g., Ashland, Butler, Huron counties; Figure 5).

### 3.2 Ohio regional surveys

**Figure 6:**
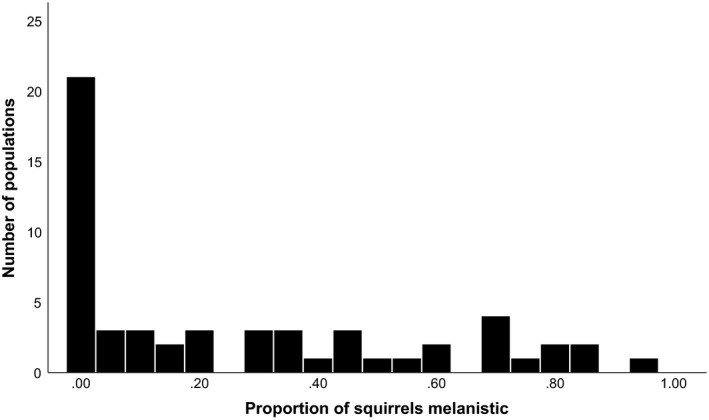
The observed range of melanism frequency in 59 eastern gray squirrel populations in Ohio (USA), surveyed in 2018 and 2019

Field surveys we conducted in Ohio in 2018 resulted in 2,542 individual squirrel observations (12,221 if the 2018 data from Wooster is included) from 59 populations. These surveys revealed a broad array of patterns in gray squirrel color morph frequency (Figure 6, Table S12) and analysis confirmed that these differences were highly statistically significant (*F* = 6.72, *df* = 11, *p* < .001). Of the 59 populations surveyed, twenty were monomorphic for the gray phenotype and 37 populations were dimorphic. Two surveyed populations consisted solely of fox squirrels. In some dimorphic populations, the melanistic phenotype was relatively rare (≤10%, four populations), in others the melanistic phenotype was reasonably common but did not represent the majority of observations (11%–50%, 19 populations). Finally, in some populations the melanistic phenotype represented the majority (>50%) of the observations (14 populations). While one population was found to be 96% melanistic in our sample, no populations were found to be fixed for the melanistic phenotype (Figure 6; Table S12). We also found a statistically significant difference in color morph frequency among habitat types (*F* = 41.92, *df* = 1, *p* < .001) with urban populations having approximately 15% more melanistic squirrels than forests, on average.

There was some tendency for nearby populations to have similar color morph frequencies. For example, all four localities in Summit county ranged from zero to 20% of the melanistic phenotype. Similarly, the three nearby localities in Medina and Lorain counties, the three localities in Knox and Richland counties and all of the more southerly populations were monomorphic. A Mantel test illustrated that there was a weak but significant positive relationship between the geographic distance between localities and the difference in their color morph frequencies (*r* = .125, *p* = .029). What seems more striking, however, is that nearby populations often differed moderately to strongly in color morph frequency. For example, Kent had 69% melanistic phenotypes while nearby Stow (<7 km away) had only 20% and Cuyahoga Falls (~11 km away) had only 3% (Table S12).

**Figure 7:**
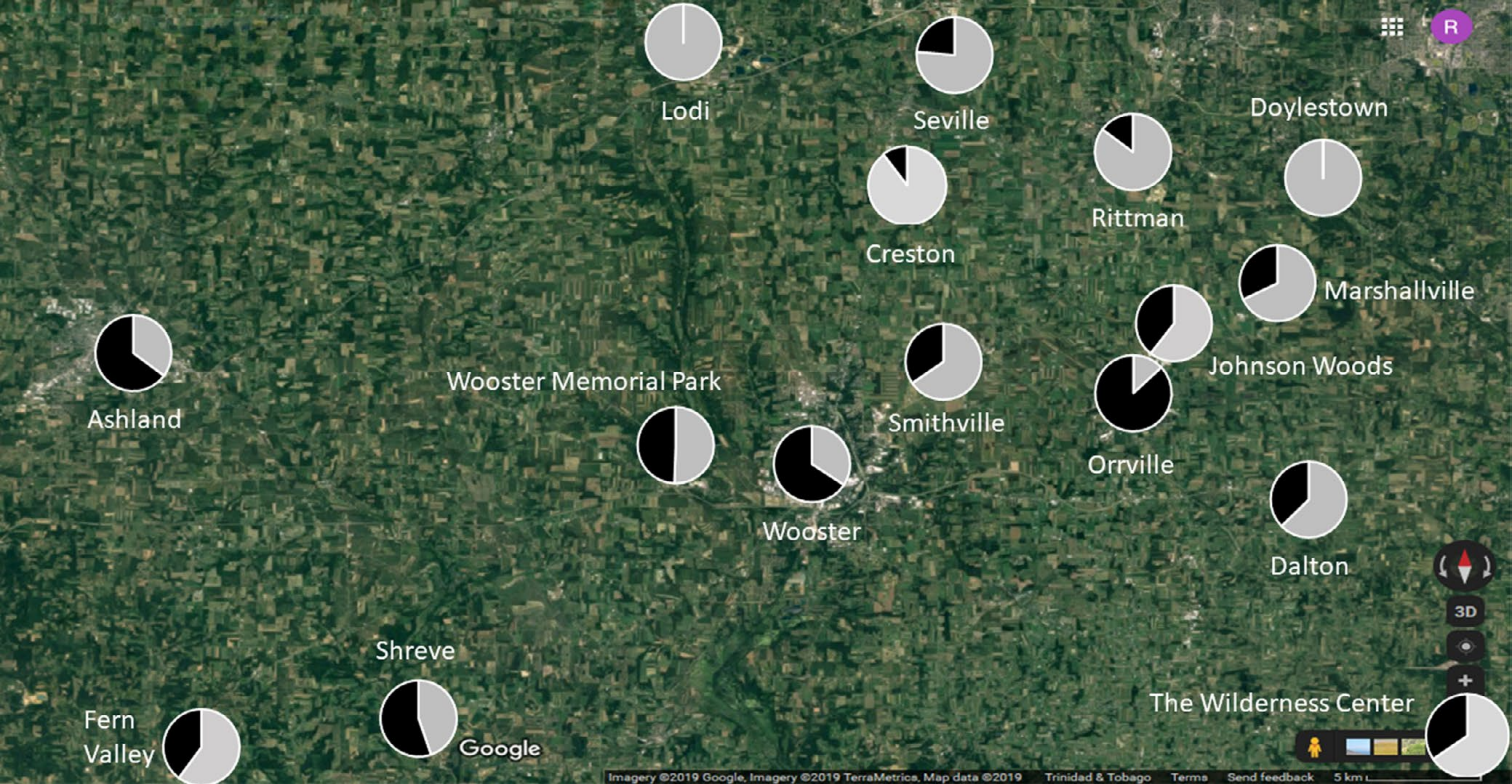
Map of the region surrounding Wooster, Ohio (USA). Each pie shows the proportion of gray morph (light shading) and black morph (dark shading) individuals observed in that population (*n* = 16). Minimum sample size = 100 squirrel observations per population. See Table S15 for full details

Field surveys in 2019 from the more intensively sampled area near Wooster resulted in 2,289 additional squirrel observations (10,332 if the 2019 Wooster data are included) averaging 153 observations per population (range: 101–509, excluding Wooster). Similar patterns were found on this smaller regional scale and statistical analysis again confirmed strong and significant differences in color morph frequency among populations (*F* = 36.95, *df* = 13, *p* < .001). The frequency of the melanistic color morph ranged from zero to 87% in these sixteen populations and neighboring populations were again sometimes strikingly different (Figure 7, Table S15).

### 3.3 Wooster surveys

**Figure 8:**
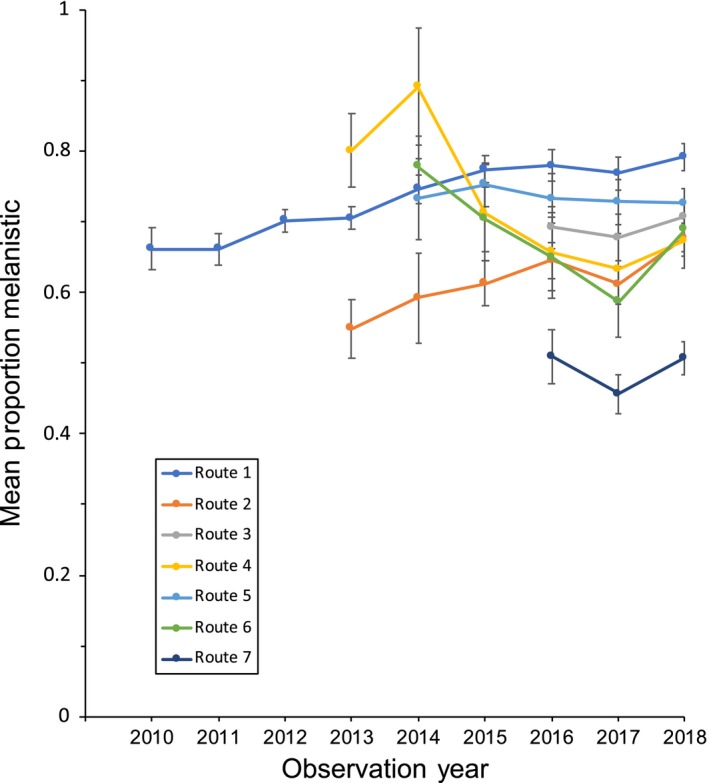
Line graph of the observed frequency of the melanistic morph (±95% confidence interval) along the seven survey routes in Wooster, Ohio (USA) from 2010 to 2018. Only route‐years that have *n* = 100 or more are shown. See Tables S13 and S14 for additional detail

During the approximately eight and a half year period between July 2010 and December 2018, 5,166 walking surveys were conducted in Wooster, Ohio resulting in 39,966 squirrel observations. The overall frequency of the melanistic phenotype in this population over that time period was 66%, when data from all survey routes and all years were pooled (Tables S13 and S14). However, there were statistically significant differences both among survey routes (*F* = 7.33, *df* = 1, *p* = .01) and among survey years (*F* = 8.97, *df* = 1, *p* = .005). There was also a significant interaction between route and year (*F* = 7.36, *df* = 1, *p* = .01). Examining the overlap of confidence intervals for the color morph frequency along individual survey routes reveals differences over very small spatial scales (Figure 8). For example, route 7 (overall average 49.7% melanistic morph) differed particularly strongly from routes 1–6 (overall average 70.1% melanistic morph) but routes 1–6 often differed from each other as well. Changes in color morph frequency over time sometimes showed consistent long‐term patterns. For example, route 1 and 2 displayed a modest but consistent incremental increase in the frequency of the melanistic morph over time (Figure 8). However, during the same time period, route 4 showed the reverse trend and others (e.g., route 3 and 5) showed little change.

## 4 DISCUSSION

While the melanistic phenotype of the eastern gray squirrel has been known for centuries (since at least the 1770s; see Mahr, 1949 also Brayton, 1882; Foster, 2000), there has been only one detailed attempt to assess its distribution and frequency quantitatively (Gibbs et al., 2019). Using nearly 13,000 citizen science records and over 50,000 field survey records, we document the spatial occurrence of this phenotype in considerable spatial and temporal detail. These data indicate that the melanistic phenotype occurs broadly throughout the Great Lakes region but can be very localized; it is absent from some areas but exceedingly common in others. Since melanism in gray squirrels is known to be a heritable trait (McRobie et al., 2014, 2009), tracking changes in the frequency of squirrel melanism over space and time is documenting the evolutionary process.

In our urban populations, we found an extremely wide range of frequencies of the color morphs (Figures 6, 7, 8). Is it possible that the strongly differing morph frequencies between populations are the result of spatially variable selection? While it is conceivable that spatially variable selection associated with abiotic (e.g., temperature) or biotic (e.g., predators)) pressures might be differentially selecting for or against the melanistic phenotype in different urban populations in our study area (thus accounting for the large among‐population differences in frequency, see Guthrie, 1967; Kiltie, 1989, 1992 for potential examples from melanistic fox squirrels), several lines of evidence argue against this view.

First, the vast majority of our sample populations from Ohio (55 of 59) were towns or cities and thus were the same basic habitat type. Our field data in urban sites were collected in residential areas and adjacent parks that consisted of human dwellings, scattered trees, and extensive lawn. These urban habitats appeared highly similar from one town to the next and likely had a similar suite of potential predators and risks associated with human‐dominated environments. Unlike in other studies where sharp habitat differences shift fitness optima abruptly (e.g., on light vs. dark substrates; Hoekstra et al., 2004), within these similar urban habitats there is not an immediately obvious basis for differential selection to occur. Gibbs et al. (2019) suggested that mortality of gray‐morph individuals may be higher in urban areas due to roadkill (if black‐morph individuals are more conspicuous and therefore avoided by motorists). However, the extremely broad range of black morph frequencies in our 55 urban study populations is not consistent with this hypothesis. It is not clear how vehicle mortality could strongly favor black‐morph individuals in some urban populations but not in others. Further, our examination of roadkill frequency in the Wooster population shows a very close match to the frequency of the color morphs in the overall population (of 44 road‐killed gray squirrels found on survey routes to date, 29 were the black morph (65.9%) and 15 were the gray morph (34.1%), RML unpublished data). On the other hand, our results agree with Gibbs et al. (2019) in that the frequency of the melanistic phenotype does significantly decrease in forests compared with urban areas (though the melanistic morph still represents 46% of all squirrel observations in forests, on average, in our data).

Secondly, if spatially variable selection were the dominant force driving color morph frequency differences among populations one would expect a much stronger clinal pattern in which nearby populations are similar to one another (McLean & Stuart‐Fox, 2014). We did detect significant spatial autocorrelation in color morph frequencies; however, this pattern was weak. In fact, we found many examples of localities that differed radically in their morph frequency from other nearby populations (Table S12). This mosaic pattern is repeated in the more intensively surveyed areas near Wooster where our color morph frequency estimates are more precise (Figure 7). Surprisingly, significant differences in color morph frequency were found even at very small spatial scales *within* populations (Figure 8). Clines can represent adaptation to temperature gradients or other environmental conditions and are commonly observed in cases where selection is the primary force influencing allele frequencies (Hoekstra et al., 2004; Kettlewell, 1958; Roulin, Burri, & Antoniazza, 2011). Our data do not suggest a strong clinal pattern.

Thirdly, our long‐term data from the Wooster population does not exhibit either consistent stability or consistent directional change but rather inconsistent patterns or idiosyncratic fluctuations over time and space (Figure 8; Table S14). Not only were there significant differences in color morph frequency among years and among survey routes but there was also a significant interaction, indicating that how the color morph frequencies changed over time depends on which route you examine. These results could be interpreted as just the same cluster of individuals driving the small‐scale spatial patterns. However, eastern gray squirrels are reproductively mature at about 10 months (Whitaker & Hamilton, 1998) and have an average life span of only about a year after birth (Barkalow & Shorten, 1973). Therefore, this dataset likely spans a substantial number of squirrel generations, making this interpreting unlikely to be wholly responsible for the patterns. Thus, since the inherent character of the patterns we documented at both large and small spatial scales is one of variation and idiosyncrasy, we suggest that genetic drift may be an important evolutionary mechanism operating in this system. This might seem unlikely with a usually rather abundant species, nonetheless, genetic drift can occur locally even in a very large population, if the population is subdivided into demes of smaller sizes (Lenormand et al., 2009; Rousset, 2013). This spatial subdivision seems likely given the small‐scale color morph frequency differences documented in the Wooster population. Further, some of our populations are villages of a few thousand people (usually surrounded by farmland); in these populations, the effective population size of gray squirrels may be rather small and the potential for genetic drift substantial.

Work on the peppered moth system has clearly illustrated the value of using color polymorphisms to track the long‐term evolutionary consequences of environmental change (Grant, Owen, & Clarke, 1996; Majerus, 1998). The patterns illustrated here are a small snapshot and continued tracking of the frequency of the melanistic phenotype in replicate populations is needed to further test the relative importance of genetic drift and other evolutionary forces in this system. If phenotypic frequencies in different populations do not change in the same directions in synchrony but rather fluctuate from year to year in idiosyncratic ways, then genetic drift will be further implicated a mechanism of evolutionary change. Consistent, long‐term changes might implicate spatially variable selection (Svensson & Abbott, 2005), while predictable oscillations would be more consistent with negative frequency‐dependent selection (McLean & Stuart‐Fox, 2014). Future work is needed to corroborate these initial patterns, to more fully assess the role of selection and to assess the importance of other evolutionary mechanisms such as gene flow and frequency dependence. What other organismal traits might be correlated with color polymorphism in gray squirrels is also in need of further examination (Ducharme et al., 1989; McKinnon & Pierotti, 2010).

The citizen science data used here expanded the geographic scope of our study far beyond what would have been possible from field surveys alone. From the standpoint of data quality, the iNaturalist citizen science data were especially valuable as each observation was objectively verifiable from a photograph (Kosmala, Wiggins, Swanson, & Simmons, 2016). However, there are clearly disadvantages of citizen science data as well. We detected significant differences in the frequencies of the color morphs in our field surveys compared with those reported by squirrel hunters. While the general patterns of spatial occurrence were roughly similar (Figure 5), we found much higher frequencies of the black phenotype than hunters generally reported. This is likely a real difference as forested environments (where hunting occurs) and urban environments (where most of our survey work occurred) tend to have different proportions of the color morphs, possibly due to differential susceptibility to predators or other sources of mortality (see Section 3; Gibbs et al., 2019). Nonetheless, uncritical pooling of these data sources would likely obscure more than it would reveal.

Another related aspect of data quality and comparability is the relatively coarse spatial scale of some citizen science data. Since relatively few observations are typically available from any given locality it is often necessary to aggregate data into larger units. This results in a loss of information since fine‐scale patterns are averaged out. This is clearly illustrated by the variation we documented both between and within populations in squirrel color morph frequency. These fine‐scale differences would not have been detected if they had been pooled with other data points from other areas. Also, these citizen science records are likely to be a biased sample as many participants might be more likely to submit observations perceived as novel. Our data lend support to this suspicion, as all of the white phenotype records (leucistic and albino squirrels) were reported by the iNaturalist database (58 of 4,678 records, 1.2%) and none (zero out of 52,840, 0%) by our surveys. Thus, using citizen science data, the real frequency of color morphs in an area is not known with high confidence since the assumption that participants submit observations of all color morphs equally is unlikely to be true. We recommend caution in making inferences based on citizen science data and especially in pooling these datasets with others. Nonetheless, the spatial scope of our work was dramatically increased with the inclusion of citizen science data and, for the right questions, it can be a very powerful approach (Chandler et al., 2017).

While typically not quantified in detail, previous workers have often stated that the black phenotype of the gray squirrel tends to be more common at higher latitudes, perhaps due to thermoregulatory advantages (e.g., Barkalow & Shorten, 1973; Brayton, 1882; Gottschang, 1981; Thorington & Ferrell, 2006). In the first large scale analysis of the geographic patterns of gray squirrel melanism, Gibbs et al. (2019), identified a moderately strong negative correlation between mean annual air temperature and probability of black morph occurrence. Thus, this supported the qualitative observations of earlier researchers. In contrast, our analysis of the relationship between latitude and frequency of melanism in gray squirrels in the Great Lakes region suggests a different pattern where mid‐latitude localities have a higher frequency of melanism on average than either low‐ or high‐latitude areas (Figure 3). This pattern seems partly driven by a relatively low frequency of melanism in Minnesota and Quebec, where several of our most well‐sampled northerly sites occurred, as well as some more southerly areas in the Great Lakes basin where melanism is common (e.g., northeastern Ohio). Future work that more comprehensively samples throughout the geographic range are needed but it is also clear that not all more northerly areas have a high percentage of melanistic individuals and some more southerly areas do. Another interesting result from the citizen science data is the documentation of the wide distribution of white phenotypes in gray squirrels. While well‐known from such places as Olney, Illinois (Barkalow & Shorten, 1973), these data demonstrate that white phenotype squirrels also occur in both Canadian provinces and in all but one of the U.S. states sampled. These include both albino and nonalbino (leucistic) phenotypes and suggest that the mutations responsible for these variants may be more common than is often supposed.

The study of color polymorphisms has a long history in evolutionary biology and despite the modern availability of tools to investigate traits that cannot be readily assessed visually, we argue that studies of color polymorphism are still quite valuable in advancing our understanding of fundamental evolutionary processes (Svensson, 2017). Furthermore, since studies of color polymorphism can, with care, take advantage of the growing data availability from citizen science efforts, we believe such studies have a bright future.

## CONFLICT OF INTEREST

None declared.

## AUTHOR CONTRIBUTIONS

RML conceived of the study. BMC, ARH, AGR, MMM, RML, and WJG conducted fieldwork, summarized data, and analyzed images. RML, BMC, and WJG conducted statistical analyses and wrote the manuscript.

## Data Availability

Data used in analyses reported in this paper are available on Dryad at https://doi.org/10.5061/dryad.zpc866t5j.
